# The SNM1A DNA repair nuclease

**DOI:** 10.1016/j.dnarep.2020.102941

**Published:** 2020-11

**Authors:** Hannah T. Baddock, Yuliana Yosaatmadja, Joseph A. Newman, Christopher J. Schofield, Opher Gileadi, Peter J. McHugh

**Affiliations:** a Department of Oncology, MRC Weatherall Institute of Molecular Medicine, University of Oxford, OX3 9DS, UK; b Structural Genomics Consortium, University of Oxford, OX3 7DQ, UK; c Chemistry Research Laboratory, University of Oxford, 12 Mansfield Road, OX1 3TA, UK

**Keywords:** Nuclease, SNM1A, DCLRE1A, Interstrand crosslink repair, Pso2

## Abstract

Unrepaired, or misrepaired, DNA damage can contribute to the pathogenesis of a number of conditions, or disease states; thus, DNA damage repair pathways, and the proteins within them, are required for the safeguarding of the genome. Human SNM1A is a 5′-to-3′ exonuclease that plays a role in multiple DNA damage repair processes. To date, most data suggest a role of SNM1A in primarily ICL repair: SNM1A deficient cells exhibit hypersensitivity to ICL-inducing agents (*e.g.* mitomycin C and cisplatin); and both *in vivo* and *in vitro* experiments demonstrate SNM1A and XPF-ERCC1 can function together in the ‘unhooking’ step of ICL repair. SNM1A further interacts with a number of other proteins that contribute to genome integrity outside canonical ICL repair (*e.g.* PCNA and CSB), and these may play a role in regulating SNM1As function, subcellular localisation, and post-translational modification state. These data also provide further insight into other DNA repair pathways to which SNM1A may contribute. This review aims to discuss all aspects of the exonuclease, SNM1A, and its contribution to DNA damage tolerance.

## 1 Introduction

DNA damage repair pathways and the proteins within them are essential for safeguarding of the genome. Unrepaired, or mis-repaired, DNA damage contributes to the pathogenesis of multiple disease states. Human SNM1A is a 5´-to-3´ exonuclease that plays a role in several DNA damage repair processes. To date, most studies highlight a role of SNM1A in ICL repair: SNM1A deficient cells exhibit hypersensitivity to ICL-inducing agents (*e.g.* mitomycin C and cisplatin) and both *in vivo* and *in vitro* experiments demonstrate SNM1A and XPF-ERCC1 function together in the ‘unhooking’ step of ICL repair. SNM1A interacts with other proteins that contribute to genome integrity outside canonical ICL repair, *e.g.* PCNA and CSB, and these may play a role in regulating SNM1A function, subcellular localisation, and post-translational modification. These interactions imply SNM1A may contribute to other DNA repair pathways. We summarise research on SNM1A and its contribution to DNA damage tolerance.

The first (and founding) member of the eukaryotic *SNM1/PSO2* nuclease family was identified in the early 1980s when genetic screens utilising *Saccharomyces cerevisiae* revealed mutant strains sensitive to bifunctional alkylating agents, but not to monofunctional alkylating agents, ionising radiation, or UV light [[1], [2], [3], [4]]. The loci mutated, *pso2* (sensitive to psoralen 2) and *snm1* (sensitive to nitrogen mustard 1), were later found to be allelic [5]. Subsequently, ten *PSO* genes were identified (*PSO1–10)*, all of which have a role in DNA damage repair; however, only *PSO2* was uniquely required for interstrand crosslink (ICL) tolerance [6]. Under normal conditions yPso2p is poorly transcribed, however, following exposure to ICL-inducing agents, its expression is increased up to four-fold [7]. *In vitro*, yeast Pso2p has been shown to possess 5´-to-3´ exonuclease activity and, possibly, structure-specific endonuclease activity [8,9].

Three vertebrate orthologues of *PSO2/SNM1* have been identified; these proteins have been denoted SNM1A, SNM1B/Apollo, and SNM1C/Artemis [[10], [11], [12]], whilst their HGNC gene names are *DCLRE1A (SNM1A), DCLRE1B (SNM1B),* and *DCLRE1C (SNM1C)* respectively. All are members of the MBL (metallo-β-lactamase) fold containing superfamily of enzymes, and can be further delineated into the self-defining β-CASP (*C*PSF, Artemis, SNM1, PSO2) family of nucleic acid processing MBLs [11,13].

Of the three human orthologues, human SNM1A (hSNM1A) has the greatest degree of sequence similarity with yPso2p; ectopic expression of hSNM1A is uniquely able to partially restore the resistance of *S.cerevisiae* bearing *PSO2* mutants to ICL-inducing agents [14,15]. It was therefore hypothesised that hSNM1A is the functional human homologue of yPso2p, and may play an analogous role in the maintenance of genomic integrity, particularly in ICL repair (keeping in mind differences in ICL repair mechanisms between higher and lower eukaryotes) [10,14].

Human SNM1B exhibits 33 % sequence identity with yPso2p in its N-terminal catalytic domain and, similarly to yPso2p and hSNM1A, possesses 5**´**-to-3**´** exonuclease activity [16,17]. Under normal cellular conditions, SNM1B is localised to telomeres *via* its interaction with the shelterin protein TRF2 (telomeric repeat-binding factor 2), where its exonuclease activity is responsible for maintaining the 5´-overhang necessary for t-loop formation at newly replicated leading strand telomeres [[17], [18], [19], [20]]. There is evidence that SNM1B plays a role in DNA damage repair, particularly in ICL repair: RNAi mediated depletion of SNM1B renders cells hypersensitive to DNA damaging agents (*e.g.* mitomycin C (MMC), cisplatin, and psoralen + UVA [16,21,22]); SNM1B co-localises with known DNA repair factors (*e.g.* MUS81, the MRN complex, and SLX4 [21,23]); loss of SNM1B is involved in defective checkpoint arrest after MMC treatment [21]; and SNM1B possesses the ability to digest DNA damage containing substrates *in vitro* [24].

Human SNM1C is a structure-specific endonuclease and is required for non-homologous end-joining (NHEJ) double strand break (DSB) repair and V(D)J recombination. Accordingly, germline mutations in *hSNM1C* result in radiosensitive severe combined immunodeficiency (RS-SCID) characterised by near-complete loss of circulating B- and T-lymphocytes, and hypersensitivity to ionising radiation (IR) [25,26]. In response to DSB formation, SNM1C is complexed to, and phosphorylated by, DNA-PK_cs_, and subsequently acquires structure-specific endonuclease activity, cleaving 5´- and 3´-overhangs, hairpins, flaps, and gapped substrates [27,28]. This activity contributes to the end-processing required to generate the necessary substrates for subsequent ligation [29]. SNM1C provides the hairpin opening activity that is required for cleaving the intermediates generated by the RAG recombinase in V(D)J recombination during antibody maturation [27]. The role for SNM1C in DNA damage repair is manifold: SNM1C depleted cells are sensitive to IR and other DSB inducing agents [30]; SNM1C is recruited (*via* DNA-PK_cs_) to sites of DNA damage [31]; and SNM1C is implicated in checkpoint maintenance and replication fork repair [[32], [33], [34]]. However, unlike SNM1A and SNM1B it seems that SNM1C does not play a direct role in ICL repair, as hSNM1C depleted cells are not sensitive to ICL inducing agents [26].

SNM1A is the subject of this review and its structural, biochemical, and cellular aspects are discussed in detail below.

## 2 Identification of the SNM1A/DCLRE1A gene

The *hSNM1A/DCLRE1A* gene (originally cloned and sequenced as Kazusa ORFeome cDNA KIAA0086), and its predicted protein product was observed to have a high degree of amino acid sequence similarity to yeast Pso2p in its C-terminal region [12]. Subsequently, analysis of the genomic organisation of the *hSNM1A* gene, and its putative protein product was performed [10]. The *hSNM1A* gene is located on chromosome 10q25.3, comprises an open reading frame of 3120 bp, consisting of nine exons, spanning from 119 bp (exon 4) to 1665 bp (exon 2). This encodes for a 1040 amino acid, 116.2 kDa protein product [10]. There have, to date, been no identified, physiologically present, *hSNM1A* splice variants. The region of sequence similarity between hSNM1A and Pso2p was mapped to the C-terminal 327 residues in hSNM1A, wherein 48 % of the amino acids were found to be identical and an additional 14 % were designated as similar [10]. This region corresponds with the ‘catalytic’ MBL and β-CASP domains of SNM1A.

Interestingly, in humans the *SNM1A* gene contains an unusually long 5´-untranslated region (UTR), containing an internal ribosome entry site (IRES), which generally suppresses translation throughout the cell cycle, the exception being, during mitosis when gene expression is upregulated. This led to the suggestion that the expression of *hSNM1A* may be temporally regulated and thus plays a role in the resolution of DNA damage that arises, is identified, or repaired during mitosis [35], although this has not been examined in detail.

## 3 Functional and structural analyses of SNM1A

As mentioned above, SNM1A is a member of the MBL structural superfamily. The ‘true’ MBLs are a subclass of the bacterial β-lactamases (BLs), responsible for antibacterial resistance by catalysing hydrolysis of all but one class (monobactams) of β-lactam antibiotics (*e.g.* penicillins and cephalosporins). The MBL family is defined by structural conservation of the characteristic α/β/β/α MBL fold. This molecular scaffold coordinates the one or two metal ions necessary for substrate hydrolysis, and most MBLs appear to bind zinc (II) in the active site, although iron (II), cobalt (II), and manganese (II) have also been reported to support catalysis [36]. MBL-fold containing enzymes catalyse a range of reactions employing a broad array of substrates, including the hydrolysis of phosphodiester bonds and thioesters, as well as redox reactions [37,38]. On the basis of biological function, the MBL enzymes have been categorised into 16 sub-groups; and the DNA and RNA processing MBLs, into groups 6 and 7, respectively. To date, nine group 6 and 7 human MBLs have been identified: SNM1A/B/C, CPSF-73, CPSF-100, ELAC1, ELAC2, Int9, and Int11 [39,40].

**Fig. 1:**
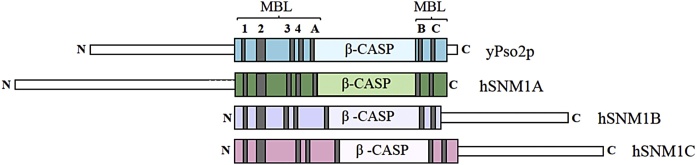
Conservation of catalytic domains between yPso2p and human orthologues. Linear representation of the amino acid sequences of yPso2p, hSNM1A, hSNM1B, hSNM1C showing the MBL and β-CASP domains with conserved motifs. The canonical MBL motifs are labelled 1–4, and β-CASP family motifs, A–C.

The active site regions of many MBL family members (which collectively catalyse diverse reactions) contain five highly-conserved motifs: where motif 1 is an acidic residue; motif 2, the HxHxDH sequence; motif 3, a histidine residue; motif 4, an acidic or cysteine residue; and motif 5, another cysteine. While these motifs are very short, they are recognisable in the context of the secondary structure; they define important active site elements, by participating in metal ion coordination and catalysis, *e.g.* hydrolysis. A subset of the nucleic acid processing MBLs is delineated into the β-CASP subfamily, including Int11, CPSF-73, and SNM1A/B/C. These members have an appended MBL domain, lacking motif 5, and an inserted β-CASP domain [13]. These conserved domains are shown in Fig. 1.

## 4 Enzymatic studies of SNM1A

Given the high degree of sequence similarity with yPso2p at the amino acid level, it was hypothesised that the hSNM1A protein may have comparable 5´-to-3´ exonucleolytic activity and initial studies confirmed this [14,41]. Two studies of purified full-length human protein derived from either yeast or insect cell expression systems confirmed hSNM1A possesses intrinsic 5´-to-3´ exonuclease activity [14,41]. This exonuclease activity was enhanced on ssDNA over dsDNA, possessed a strict requirement for a free 5´-phosphate group, and was abolished by a D736A substitution (motif 2 in the MBL domain) [14,41].

A follow-up study by Sengerová and colleagues utilising recombinant (insect cell-derived) truncated hSNM1A, containing the catalytic MBL and β-CASP domain core, demonstrated that the addition of several divalent metal ions (CaCl_2_, MnCl_2_, or MgCl_2_) stimulated enzymatic activity; these metal ions seem to be required in addition to the ‘intrinsic’ active site metal ions. ZnCl_2_, NiCl_2_, and CoCl_2_ did not stimulate enzymatic activity and were inhibitory at concentrations greater than, or equal to, 0.1 mM. The addition of exogenous MgCl_2_ had the greatest effect on stimulating nuclease activity, although the reasons for this remain unclear, as does the identity of the metal ions used by hSNM1A *in vivo*. Conversely, the addition of chelating agents (ο-phenanthroline, EGTA, or EDTA) abrogated activity, as did mutations in putative metal ion-coordinating residues (D736A/H737A). hSNM1A was also shown to bind and digest an array of substrates and, importantly, was able to digest past a site-specific ICL [24]. This was an important observation as it reconciled the cellular phenotype (where loss of SNM1A is characterised by sensitivity to ICL inducing agents) with the *in vitro* biochemical activity. The pathways and interactions contributing to this process are discussed in detail below.

Whether hSNM1A functions as an endonuclease is a point of some contention. Two studies reported that when the availability of 5´-ends is blocked by the presence of a biotin molecule, or hydroxyl group, the activity of hSNM1A is abrogated, thus suggesting no intrinsic structure-specific endonucleolytic activity; this was observed on dsDNA, cross-linked, flap, and hairpin substrates [24,41]. Conversely, a third study identified endonucleolytic activity of hSNM1A on regions of single-stranded DNA in replication, or repair, intermediate structures; for example, flaps, overhang, gaps, bubbles, and loops. *In vitro* nuclease assays showed that hSNM1A was able to make multiple endonucleolytic incisions on a fork-ICL substrate on the single stranded region 5´ to the ICL, and then exonucleolytically process past the ICL [15]. Differences in enzyme and substrate concentrations may reconcile these apparently contradictory results. Sengerová et al. used 4 nM hSNM1A in radiolabelled nuclease assays with a crosslinked substrate, whereas, Buzon et al. used 180 nM, and each used 100 nM of substrate, for an incubation period of up to 120 min [15,24]. Therefore, it may be that at lower concentrations hSNM1A functions solely as an exonuclease, but at higher concentrations the activity has reduced specificity and some endonucleolytic processing may be observed.

## 5 Structural studies of SNM1A

**Fig. 2:**
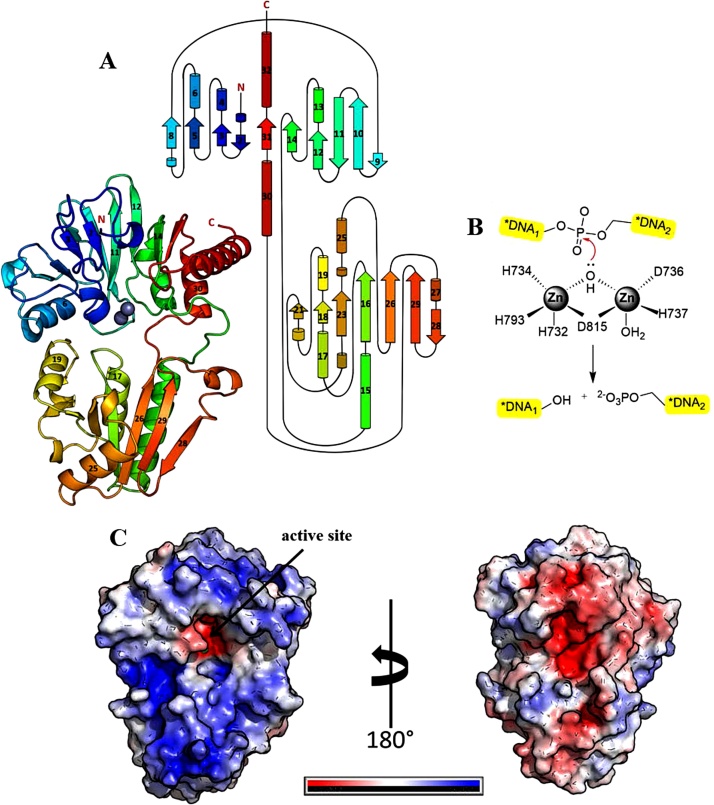
Structural features of hSNM1A. A: cartoon representation and topology map of hSNM1A, coloured from blue (N-terminus) to red (C-terminus). The MBL and β-CASP domains are as indicated, with the contributing α-helices and β-sheets as labelled. The active site metal ions are depicted as grey spheres, and the N- and C-termini are as labelled. B: outline mechanism for SNM1 family enzymes; note metal ion use by hSNM1A *in vivo* is uncertain. C: the electrostatic surface potential of hSNM1A shown from two orientations, where red is more electronegative, and blue more electropositive. The active site is indicated.

A structure of the MBL/β-CASP domain of hSNM1A (698–1040) has been solved, both in the apo form, to 2.19 Å (PDB: 5AHR [42]), and with inhibitors bound in the active site (PDB: 5NZW and 5NZY, to 2.7 and 1.5 Å, respectively). These structures encompass the catalytic core of the protein and reveal several distinctive features. The topology fold of the hSNM1A MBL domain is typical to other members of the superfamily and comprises a four-layered β-sandwich (α/β-β/α), with two mixed β-sheets, flanked by two α-helices on either side. The β-CASP domain consists of a four-stranded parallel β-sheet, flanked by three α-helices on one side, one on the other, and is inserted between strands 10 and 11 of the MBL domain [42]. The structure of hSNM1A is represented in Fig. 2.

The crystal structures provide insight into the hSNM1A active site and potential mode of DNA binding and suggest a mechanism, based on that for the true MBLs (Fig. 2) [24]. In the outlined hSNM1A mechanism, two metal ions are positioned within the active site, and the central water coordinated between them is proposed to be activated as a hydroxide ion, and can thus act as a nucleophile to attack the phosphodiester bond, resulting in exonucleolytic cleavage of the terminal nucleotide; one of the phosphate oxygens of the scissile phosphodiester may also coordinate to one of the active site metal ions. However, whether hSNM1A requires one or two metal ions for catalysis is incompletely understood. The crystal structure of the apo version of hSNM1A reveals one zinc ion (coordinated by the side chains of D815, H734, H732, H793) in the first metal ion-binding site (PDB: 5AHR and [42]). These precise contacts, and the geometry of the active site are entirely conserved with the hSNM1B structure (PDB: 5AHO and [42]) and the recently solved hSNM1C structure (PDB: 6TT5). In the case of hSNM1B the octahedral coordination of the first zinc ion is completed by the carboxyl and hydroxyl oxygens of a buffer-derived tartrate molecule. This is comparable to what has been observed in an analogous structure for hSNM1A, where the coordination of the first metal ion (in this structure Ni, though this is unlikely to be an *in vivo* metal ion) is completed by two carboxyl oxygens of a malonate molecule from the crystallisation buffer (PDB: 5Q2A, 1.5 Å resolution). Thus, it may be that for hSNM1A, when substrate binding occurs, the coordination network of the first metal ion is completed by the presence of the nucleophilic water molecule, and the phosphodiester backbone of the DNA substrate. However, this proposed mechanism remains speculative, and future structural studies, particularly with a DNA substrate(s), should elucidate this further.

**Fig. 3:**
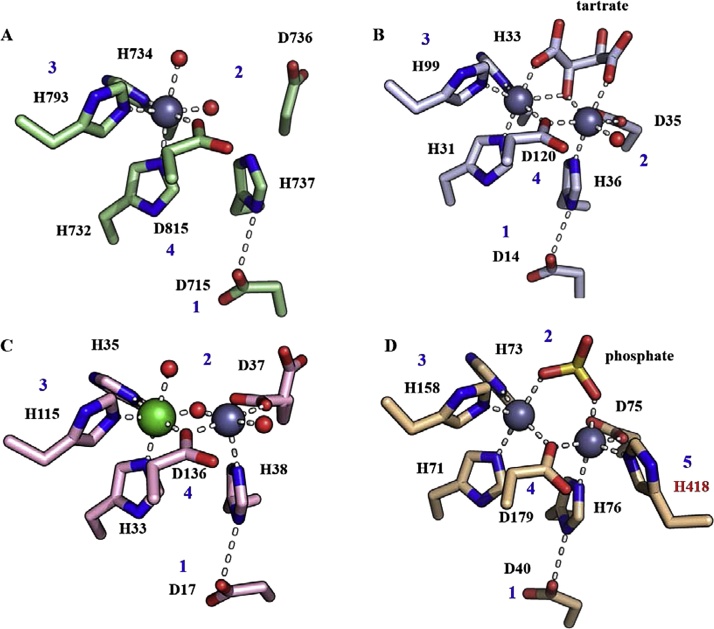
Comparison of the active site architectures of DNA- and RNA-processing β-CASP/MBL family members. A) hSNM1A (PDB: 5AHR); B) hSNM1B (PDB: 5AHO); C) hSNM1C (PDB: 6TT5); D) CPSF73 (PDB: 217 T). The conserved MBL motifs are in blue, and the additional histidine (motif 5) in CPSF73 is red. The active site Zn^2+^ ions are grey spheres, and the Ni^2+^ ion in the hSNM1C structure is in green. Water molecules are small red spheres.

The second potential metal ion-binding site in hSNM1A is unoccupied in all reported crystal structures; however, all residues that coordinate the second metal ion (also octahedrally) in both the hSNM1B and hSNM1C structures are conserved in hSNM1A (D35, D736, and H737) [42]. Structures of RNA-processing β-CASP MBLs; RNase J1 [43], RNase J [44,45], CPSF-73 and CPSF-100 [46] have all been solved with two Zn^2+^ ions coordinated in the active site. However, it is notable that the aforementioned RNA-processing β-CASP MBLs have one more histidine coordinating residue at the second metal ion-binding site, a feature that is not conserved in the DNA-processing β-CASP MBLs, and therefore it seems quite possible that hSNM1A binds a second metal less tightly. For a comparison of the active site architecture between the DNA and RNA processing β-CASP MBLs, see Fig. 3. Nevertheless, it seems plausible that the di-metal form of hSNM1A is more catalytically active, as this may optimally coordinate and activate the water molecule for phosphodiester hydrolysis; accordingly the D736A variant of SNM1A is catalytically inactive [24,42]. The identity of the catalytically relevant (or, indeed, inhibitory) metal ion(s) for hSNM1A, at this stage thus remains to be elucidated, although use of two Zn (II) ions would be consistent with proposals for other β-CASP MBL family members.

Perhaps the most striking feature of the hSNM1A crystal structure is the electrostatic surface charge distribution. From the perspective of the active site, hSNM1A is highly positively charged (Fig. 2); this is mostly due to the side-chains of a number of lysine and arginine residues located on both MBL and β-CASP domains [42]. Current hypotheses suggest that this charged face interacts with the negatively charged phosphodiester DNA backbone and enhances substrate binding, in addition to the specific contacts likely made in the active site, in a non-sequence-specific manner. This positively charged, putative ‘DNA binding groove’ is suggested to mediate the enhanced processivity that SNM1A manifests with higher molecular weight DNA, as well as facilitating binding to substrates that are bulky and which may not be well accommodated in the active site (*i.e.* those that contain DNA damage) [42]. Accordingly, mutating two adjacent residues at one end of the proposed DNA binding groove had no effect on the catalytic turnover or efficiency, though processivity on plasmid DNA substrates, and the capacity to digest past an ICL-containing dsDNA substrate was markedly reduced. However, it was noted that other mutations within this region had a much less pronounced effect [42].

## 6 Mouse studies into the function of SNM1A

To date there are three reports examining the effects of mSNM1A disruption in mice, with sometimes inconsistent results. The first, generated from the 120/SvJ background, involved deletion of intron 3 and exon 4 of the *mSNM1A* gene, and subsequent loss of most of the catalytic motifs. These mice were viable, developed normally, exhibited no major defects, and were fertile. However, after treatment with MMC, survival rates indicated an enhanced sensitivity when compared with WT mice, but not to other DNA damaging agents, including 8-methyoxypsoralen + UVA, MMS, melphalan, UV_254 nm_, or cisplatin [47]. Similarly, in a 2008 study, *mSNM1A* knockout mice from the same genetic background (120/SvJ), wherein exon 2 was disrupted, exhibited normal development, fertility, and life expectancy [48]. Interestingly, when exons 2–7 were disrupted to generate *mSNM1A* null animals from a different genetic background (C57BL) a more complex phenotype was observed, characterised by decreased life expectancy and increased rates of tumourigenesis. When there was dual loss of *p53* and *mSNM1A*, rates of tumourigenesis were enhanced, suggesting that in the absence of *mSNM1A* genome damage that is countered by the tumour-suppressing activities of *p53* accumulates. Loss of *mSNM1A* in a *FancD2^−/−^* background also lead to semi perinatal lethality, suggesting that mSNM1A contributes to DNA repair, and particularly ICL repair, outside the canonical Fanconi Anaemia (FA) repair pathway. *mSNM1A* null mice exhibited hypersensitivity to the spindle poisons, nocodazole and Taxol, further suggesting a role for mSNM1A in maintaining genomic integrity. In male *mSNM1A^−/−^* mice an increase in bacterial infections was also reported, contributing to increased mortality, although the reason for this is currently unclear [49].

## 7 The cellular function of SNM1A

Most attempts to understand the role of SNM1A in higher eukaryotes have focussed on cellular studies, and whilst these have identified a strong contribution of SNM1A to DNA repair, fully elucidating its function has proved challenging. This is attributable, at least in part, to low levels of endogenous protein expression, and the cytotoxicity of SNM1A over-expression in mammalian cell systems [35,41,47,50]. Nevertheless, the current understanding of the cellular activities of SNM1A is summarised below.

mSNM1A contains a nuclear localisation sequence (NLS) and localises to the nucleus in mouse ES cells [47]. In MEFs, ES cells, or skin fibroblasts, loss of *mSNM1A* result in sensitivity to MMC when compared with isogenic cell lines, but not to other DNA damaging agents (including IR, 8-methoxypsoralen + UVA, MMS, UV_254 nm_, and melphalan), in clonogenic survival assays [[47], [48], [49]]. After treatment with MMC, *mSNM1A^−/−^* MEFs also exhibited increased chromosomal instability, characterised by increased breaks and radials [48]. Similar to the organismal phenotype, *mSNM1A^−/−^* MEFs were hypersensitive to treatment with the spindle poisons, nocodazole and Taxol [49,51]. After exposure to nocodazole and Taxol, *SNM1A^−/−^* MEFs also exhibited failure to arrest at the prophase-to-metaphase transition checkpoint, resulting in increased micronuclei, polyploidy, and decreased cellular viability. This implicates mSNM1A in maintaining genomic integrity through checkpoint monitoring [51].

To probe the cellular networks in which mSNM1A participates, functional relationships between mSNM1A and other known DNA repair factors were examined, particularly with regards to ICL repair. The formation of RAD51 foci, after induction of DNA damage, is used to indicate the formation of synapsed intermediates, generated by recombination-mediated repair of DSBs, in downstream steps in ICL repair, and during the protection and remodelling of damaged replication forks [52,53]. Loss of *mSNM1A* in MMC-treated ES cells did not affect RAD51 foci formation, implying that mSNM1A either functions downstream of RAD51, functions in a parallel repair pathway, or that there is redundancy with another repair factor [47].

Similar to mouse data, loss of SNM1A in chicken DT40 cells resulted in sensitivity to MMC and cisplatin; this sensitivity could only be overcome by provision of a functionally intact MBL domain. Interestingly, the double disruption of *chSNM1A* and *chSNM1B* enhanced sensitivity when compared with the respective single-gene disruptants, suggesting functionally overlapping or parallel roles for these genes in ICL repair. Further investigation into the genetic relationship of *chSNM1A* with other repair factors in DT40 cells found that *chSNM1A* is non-epistatic with *XRCC4* (an XLF-paralogue, involved in NHEJ), *RAD18* (an E3 ubiquitin ligase that regulates TLS) and *FANCC*. This led to the conclusion that chSNM1A has, at least partially, distinct functions from the canonical HR, TLS, or FA DNA repair pathways, respectively [54].

Another study utilising chicken DT40 cells observed that *chSNM1A^−/−^* cells were resistant to high doses of etoposide, and that chSNM1A appears to play a role in mediating etoposide-induced apoptosis. A similar phenotype was observed for each of the chSNM1 nucleases (SNM1A, SNM1B, SNM1C), and they appeared to function at least partly in parallel, or have overlapping functionality from one another, as concomitant depletion led to an additive phenotype. The specific role that chSNM1A (or chSNM1B/SNM1C) plays in this pathway is unclear; however, the data suggest that its involvement is upstream of caspase activation, and that it may be involved in mediating the cleavage of DNA during the apoptotic process [55].

With regards to human cells, there have been a number of studies defining the subcellular localisation of hSNM1A, its interaction and functional relationships with other repair factors, its role in cell-cycle checkpoints, and the effects of post-translational modifications (PTMs). As observed with mouse and chicken DT40 cells, hSNM1A appears to be poorly expressed and localises to the nucleus. Overexpressed hSNM1A localises to either large, amorphous nuclear structures (approximately 2 μm in diameter), referred to as ‘SNM1 bodies,’ or to multiple smaller foci, and which of these predominates appears to be influenced both by cell cycle stage, and DNA repair status. For example, after treatment with 10 Gy IR in MCF7 cells, there was an increase in ‘hSNM1A repair foci’ and a concomitant decrease in the ‘SNM1 bodies’ [50]. It is hypothesised that the presence of these ‘SNM1 bodies’ are simply a sequestration of insoluble hSNM1A protein, as overexpression was previously shown to be toxic in mammalian cells [35,41,47,50,54].

After treatment with IR, these hSNM1A containing foci were shown to co-localise with known DSB repair factors, MRE11, 53BP1, and BRCA1 (albeit more weakly), but not BRCA2 or RAD51 [50,56]. Both MRE11, as part of the MRN (MRE11-RAD50-NBS1) complex, and 53BP1 are rapidly recruited to DSBs, where they mediate downstream repair processes [[57], [58], [59], [60]]. hSNM1A was shown to co-localise with 53BP1 both before and after DNA damage, and 53BP1 was shown to co-immunoprecipitate with hSNM1A in untreated cells, although 53BP1 was not necessary for the recruitment of hSNM1A to DNA damage repair foci [50]. Instead, the recruitment of hSNM1A to DNA damage repair foci in response to IR was found to be dependent on the activity of the ATM kinase. Following from this, hSNM1A was shown to be a target for ATM phosphorylation *in vitro*, and to be required for the G_1_-checkpoint arrest following IR treatment [56]. The exact mechanism by which hSNM1A is recruited to subnuclear foci is unclear, although the data suggest that this is dependent on a functional MBL domain, as point mutations in this region (H994A, D838 N) largely abrogate foci formation [54]. Interestingly, these two residues are located proximal to the active site, and there may be a link between catalytic activity and foci formation (though it is possible these mutations alter the protein fold stability).

hSNM1A has also been shown to interact and colocalise with the SUMO E3 ligase, PIAS1 [54]. Similar to ubiquitination, SUMOylation can affect protein stability, subcellular localisation, and protein-protein interactions [61]. However, it unclear whether hSNM1A is a substrate for PIAS, either *in vitro* or *in vivo.* It is notable that H994 and D838 were important for the interaction of PIAS1 with hSNM1A [54], raising the possibility of PTMs affecting subcellular localisation, or regulating enzymatic activity.

Another PTM associated with initiation and regulation of DNA repair pathways is PAR-ylation, as catalysed by PARPs (poly(ADP-ribose)-polymerases) [62]. Pattern recognition and homology searches identified a putative PBZ (PAR binding zinc finger) motif within hSNM1A, although this has not yet been validated experimentally, and the role of this in mediating the activity of hSNM1A in response to DNA damage remains to be elucidated [63].

Further to this, an *in silico* pattern recognition search identified a highly conserved putative PIP (PCNA interacting protein) box and a UBZ (ubiquitin binding zinc finger) within the primary amino acid sequence of SNM1A. These were confirmed experimentally *in vitro*, and within cells hSNM1A was shown to physically interact with PCNA *via* its PIP box. When PCNA was ubiquitinated in response to DNA damage, this interaction was enhanced, as the UBZ of hSNM1A was able to make an additional point of contact. This interaction of hSNM1A with PCNA (*via* the PIP box) was absolutely required for hSNM1A foci formation, both in unstressed and stressed conditions. However, DNA damage (treatment with MMC or UV) was required to activate the UBZ-dependent assembly of hSNM1A, and this, in turn, was dependent on the presence of RAD18 [64]. In response to replication fork stalling, RAD18 is an E3 ligase that is able to ubiquitinate PCNA, triggering downstream repair and damage tolerance pathways [65,66]. Therefore, a model was proposed whereby hSNM1A interacts with PCNA at replication forks *via* its PIP box, and, in cases of replication fork stalling at damage, RAD18-mediated ubiquitination of PCNA enhances the interaction between PCNA and hSNM1A, thus promoting the formation of repair foci and initiating downstream repair pathways [64].

More recent studies examining the replication fork proteome in both human cells and *Xenopus* egg extracts identified the presence of SNM1A at replication forks, both in unstressed conditions, and after treatment with DNA damaging agents. Two of these studies utilised iPOND (isolation of proteins on nascent DNA), coupled with mass spectrometry [67,68], and the third utilised chromatin mass spectrometry (CHROMASS) in *Xenopus* plasmid assays [69]. These found hSNM1A present at the replication fork in unstressed conditions, showed damage-specific enrichment after treatment with hydroxyurea [67], and also accumulated at sites of psoralen ICLs [69].

The role of hSNM1A has also been explored in the context of ICL repair and, as with chicken and mouse cells, human cells deficient in SNM1A exhibit sensitivity to MMC [48,54,70]. Again, similarly to chSNM1A and mSNM1A, hSNM1A appears to function in ICL repair outside the canonical FA repair pathway, or downstream of FA core complex recruitment and processing. Depletion of hSNM1A in *FANCA* deficient fibroblasts revealed a nonepistatic relationship after treatment with MMC, and depletion of hSNM1A alone did not affect FANCD2 monoubiquitination in response to ICL inducing agents [48]. Interestingly, survival assays after treatment with cross-linking agents, showed epistasis between hSNM1A and the structure-specific endonuclease, XPF-ERCC1 [70]. The functional relationship between hSNM1A and XPF-ERCC1 in collaborating to process ICLs will be discussed in more detail below.

The relationship between hSNM1A and other DNA repair nucleases is of interest. Human FAN1 (FANCD2 associated nuclease) has structure specific endonuclease activity on 5´-flap substrates and 5´-to-3´ exonuclease activity on ssDNA (albeit releasing 3 nucleotide reaction products). In cellular studies, hFAN1 is required for resistance to ICL-inducing agents [71], and, *in vitro,* hFAN1 can incise either side of an ICL embedded in dsDNA with a 5´ flap, thereby effectively unhooking it [[72], [73], [74]]. Interestingly, hFAN1 can function both within, and independently from, the canonical FA-pathway in ICL repair. Double disruption of *mSNM1A* and *mFAN1* resulted in increased sensitivity to MMC treatment, when compared with either disruptant alone, suggesting that these two enzymes function (at least partially) independently to facilitate ICL unhooking [71]. A more recent study suggested that hSNM1A may act in the same pathway as the DNA repair exonuclease, SAN1, after treatment with MMC. A similar epistatic relationship was observed between SAN1 and FAN1, and it is possible these nucleases function together to resolve ICLs in an FA-independent manner [75].

There is also some evidence that hSNM1A plays a role in replication-independent ICL repair. In dividing cells, ICLs are repaired when one, or two, replication forks collide with the ICL [76,77]. However, there is evidence of alternate repair pathways that occur outside S-phase in replicating cells, or in post-mitotic cells. The data suggest that replication-independent repair involve factors from either transcription-coupled (TC), or global-genome (GG) NER (nucleotide excision repair) pathways [78,79]. One such factor is CSB, a TC-NER protein, implicated in the repair of MMC, cisplatin, or psoralen/UVA induced interstrand crosslinks in G_0_/G_1_ phases of the cell cycle [[80], [81], [82], [83]]. Interestingly, a yeast two-hybrid screen and co-immunoprecipitation experiments revealed that CSB physically interacts with hSNM1A, and confocal microscopy showed they colocalise to trioxsalen + laser-induced ICLs in cells [83]. This, of course, raises the possibility that hSNM1A is able to contribute to ICL-processing outside of the S-phase dependent (FA mediated) ICL repair pathway.

**Fig. 4:**
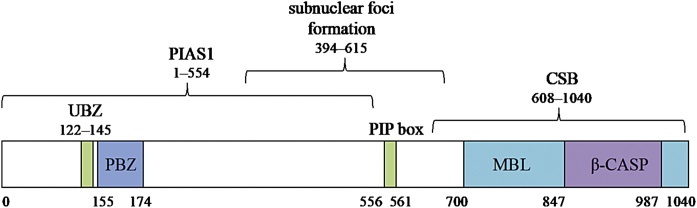
Linear representation of hSNM1A showing domain boundaries and interacting regions. UBZ = ubiquitin binding zinc finger, PIP box = PCNA interacting motif, both from [64]. PBZ = PAR binding zinc finger, from [63]. MBL and β-CASP from [42]. The PIAS1 and CSB interactions were not mapped specifically, but were within depicted regions, from [54] and [83] Iyama et al., respectively. Subnuclear foci formation, within this region, from Richie et al.

Whilst the C-terminal region of hSNM1A contains the catalytic domains, the N-terminal region appears to be important for mediating protein-protein interactions, containing sites for PTMs, and directing foci formation. These regions and important features are summarised in Fig. 4.

## 8 A mechanistic understanding of SNM1A in DNA damage repair

A number of *in vitro* biochemical reconstitution experiments have provided additional insight into the role of hSNM1A in ICL repair. The first of these was the observation that recombinant, truncated hSNM1A (698–1040), as well as full-length hSNM1A, possess the ability to digest past a site-specific crosslink *in vitro* [24]. There have also been efforts to partially reconstitute ICL repair pathways *in vitro*, to elucidate the molecular steps to which hSNM1A may contribute.

**Fig. 5:**
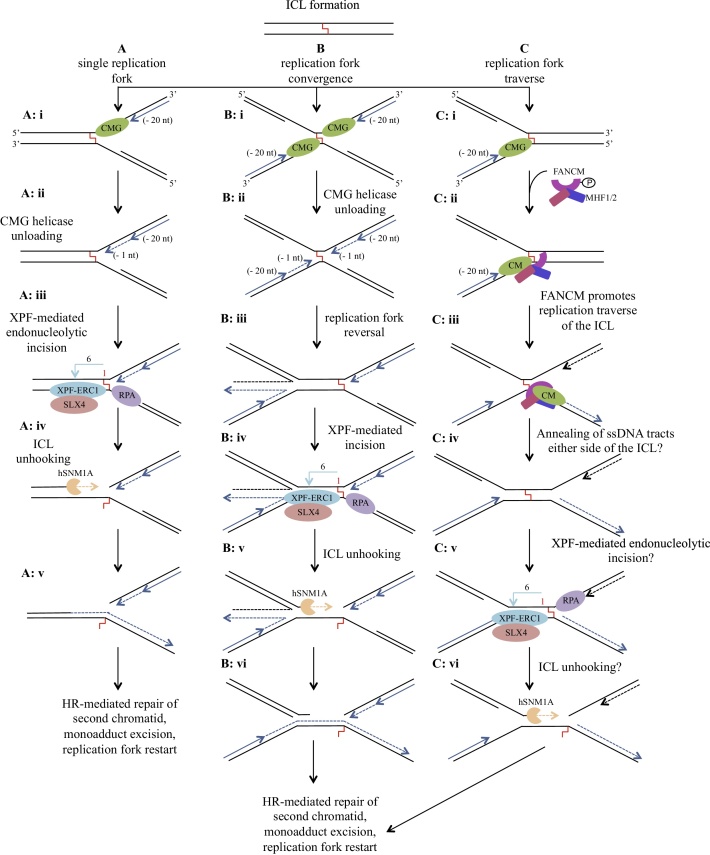
Suggested roles for hSNM1A in replication-coupled ICL repair. In these models the collaborative activity of hSNM1A, XPF-ERCC1, RPA is able to facilitate ICL unhooking. A: a single replication fork encountering an ICL, stalls ∼20 nt away (i) [92], this is followed by CMG helicase unloading and progression of the replication fork to the ICL (−1 nt) (ii) [94]. In the presence of RPA, XPF-ERCC1 is able to incise 6 nt 5´ to the ICL within the duplex region (iii), hSNM1A is then able to load onto this nicked substrate and digest past the ICL, thus unhooking it (iv) [70,97]. The digested strand then becomes a substrate for TLS polymerases, replication fork restart, and HR-directed repair of the broken chromatid. The tethered single nucleotide is likely removed by NER (v). B: in the case of dual fork convergence at an ICL, each leading strand replication fork stalls ∼20 nt away (i), before there is CMG unloading at each fork [76], allowing each of the leading strands to progress the ICL (−1 nt) [77] (ii). One fork then undergoes replication fork reversal [95] (iii), generating a tract of dsDNA, which becomes a substrate for XPF-ERCC1 incision [95] (iv), hSNM1A lesion bypass (v), and repair is able to proceed as in A (vi). C: a single replication fork encounters an ICL (i) and, *via* the recruitment and translocase activity of FANCM/MHF1/2 (ii), is able to bypass the lesion and continue replication 3´ to the ICL (iii) [91,100]. The following steps are purely speculative, but it is possible that ssDNA tracts surrounding the ICL undergo spontaneous reannealing (iv), and provide a substrate for XPF-mediated incision (v) and hSNM1A unhooking (vi). Nevertheless, alternative scenarios may be imagined, especially in the absence of significant dsDNA character around the traversed ICL, where alternative endonucleases (*e.g.* SLX1, MUS81-EME1/2) could make the first incision as an entry point for hSNM1A. Schematic information: dark blue solid arrows represent initial approach by nascent leading strands, and dotted arrows represents progression that occurs after initial fork stalling. Lagging strands are indicated in black, and dotted arrows again represent progression that occurs after initial fork stalling. The light blue arrows represent incision by XPF-ERCC1, and the peach dotted arrow, exonucleolytic digestion by hSNM1A. Figure adapted from [91,95,97].

There are presently three main models for replication-coupled ICL repair, which have been comprehensively reviewed elsewhere, and readers seeking a detailed account of these processes are directed to [[84], [85], [86], [87], [88]]. In summary, these are: collision of a single replication fork with an ICL [89]; dual collision of converging replication forks [77,90]; and replication fork traverse past the ICL mediated by FANCM’s translocase activity [91]. These models have largely been generated from experimental data utilising either mammalian cell biology methods or *Xenopus* egg extracts examining the repair of plasmids containing site-specific ICLs [77]. The steps and repair intermediates generated for each of these models are summarised in Fig. 5. Currently, the dual collision replication fork model predominates, whereby each nascent leading strand stalls ∼20–40 nt from the ICL, due to steric constraints by the CMG helicases [92]. One, or both, of the CMG helicases is then unloaded in a TRAIP-dependent manner [93], allowing one replication fork to proceed to the site of the ICL [94], where it may undergo replication fork reversal [95]. This regressed fork structure then becomes a substrate for nucleolytic incision and subsequent ICL-unhooking. A number of nucleases have been implicated in this incision and unhooking process: XPF-ERRC1, MUS81-EME1, SNM1A, SNM1B, FAN1, and SLX1; however, at this point the data suggest that XPF-ERCC1 is indispensable for this process [96].

Following from these models, biochemical results demonstrate that XPF-ERCC1 and hSNM1A can collaborate to very efficiently incise and unhook the ICL [70,97]. It was shown that on a structure mimicking a stalled replication fork at an ICL, XPF-ERCC1 is able to endonucleolytically incise 5´-to the crosslink, in the presence of RPA. hSNM1A was then able to load onto the crosslinked substrate at the incision point and then process past the ICL, leaving a drug-tethered single nucleotide residual adduct, effectively unhooking it [70]. It is likely that this processed ICL moiety is a substrate for downstream TLS and homologous recombination (HR) directed repair and subsequent excision of the monoadduct. Importantly, in this system, the collaborative activity of XPF-ERCC1-RPA and hSNM1A would be capable of acting on intermediates generated by both a single and dual/converging fork ICL collision [97]. The endonucleolytic activity of XPF-ERCC1 requires a short stretch of dsDNA, therefore the observation that fork regression has been observed following replication fork encountering the ICL is pertinent [95]. In cases of FANCM-mediated ICL traverse, the structure of the post-replicative ICL-containing DNA is not known; however, it is likely that this may also be a substrate for XPF-ERCC1-RPA and hSNM1A nucleolytic processing (Fig. 5). It is important to note that the experiments validating FANCM-mediated ICL-traverse were performed on psoralen lesions, which are also able to be unhooked through the glycosylase activity of NEIL3 [98,99], thus raising the possibility that ICL-traverse could be linked to that pathway, which may not be relevant to other forms of ICLs (*e.g.* platinums, nitrogen mustards, aldehydes).

In the context of replication-independent ICL repair, it was shown that the presence of CSB stimulates the nuclease activity of hSNM1A *in vitro* [83]. CSB is suggested to play a role in chromatin remodelling and is part of the SWI12/SNF2 family of DNA-dependent ATPases [101]. Interestingly, in the *in vitro* system used in this study, CSB stimulated the activity of hSNM1A on DNA substrates that it, itself is unable to bind, thus suggesting this stimulation arises due to protein-protein interactions [83].

Taken together, the contents of this review suggest an important role for hSNM1A in ICL processing and repair. The exact mechanism remains to be elucidated, and there are still many outstanding pieces of the puzzle to fit. For example: why is there sensitivity for some cross-linking agents (MMC or SJG-136), over others (cisplatin); and, to what extent is there redundancy or synergy with other repair nucleases (FAN1 or hSNM1B)? These questions provide important avenues for further research.

The potential role for hSNM1A is DSB repair is less clear. hSNM1A depleted cells do not display overt sensitivity to IR, but hSNM1A foci do form in response to IR [56], and colocalise with known DSB repair proteins [50]. hSNM1A is also required for DSB-induced cell-cycle arrest [50]. Again, further delineating this is an area for future research.

## 9 SNM1A in disease

Cellular data show hSNM1A is important for the maintenance of genome integrity, and the phenotype of the SNM1A knockout mice led some to conclude that mSNM1A functions as a tumour suppressor [49]. A number of genetic studies have also associated SNPs in the *hSNM1A* gene as being associated with cancer risk. One SNP (*rs3650898*) resulting in the coding variant, H317D, was marginally associated with increased small cell lung carcinoma risk [102]. This same SNP was also significantly associated with the development of peripheral neuropathy after treatment with oxaliplatin-fluoropyrimidine chemotherapy, in an analysis of 2183 patients with advanced colorectal cancer [103]. Another SNP, (*rs41292634*) resulting in a nonsense substitution in exon 2, was associated with an elevated incidence of cancer in a BRCA1 and BRCA2 negative population with hereditary breast cancer risk [104].

Expression levels of *hSNM1A* also appear to be associated with both the process of tumourigenesis and survival outcomes. In human cells, SNM1A is ubiquitously expressed at very low levels, with higher levels of expression in the brain, testes, and thyroid [50,105,106]. Interestingly, in individuals with ovarian cancer *hSNM1A* was identified as one of two 7-gene functional groups, where elevated transcript expression was associated with decreased survival [107]. Furthermore, in analyses of 47 individuals with colorectal cancer, *hSNM1A* transcript expression was significantly elevated in tumour tissue compared with control mucosa [108]. Changes in transcript expression of *hSNM1A* in cases of colorectal cancer are a potential point of interest. Colibactin was recently identified as genotoxin directly capable of causing ICLs [109,110], and colibactin expressing bacteria are associated with the pathogenesis of colorectal cancer [111]. Whether increases in *hSNM1A* transcript expression is related to colibactin-exposure and/or subsequent ICL induction remains an intriguing question.

## 10 Targeting SNM1A as a therapeutic strategy

Additional research into the relationship between *hSNM1A* gene expression and tumourigenesis will provide greater insight as to whether increased protein expression is associated with tumourigenesis and disease progression. However, these preliminary insights, in addition to the therapeutic tractability of targeting DNA damage repair pathways [112,113], have led our lab, and others, to explore the development of small molecule inhibitors of hSNM1A. Many chemotherapeutic agents function by inducing cytotoxic DNA damage, however, over time tumour cells become resistant, in part by upregulation of DNA repair pathways [114]. In addition, many tumour cells have lost some DNA repair functionality and therefore rely solely on redundant or alternate pathways. Therefore, inhibition of DNA damage repair factors (such as hSNM1A) may be useful as adjunctive chemotherapeutic agents, or in tumour-specific cases of synthetic lethality. Interestingly, the cephalosporins, cefotaxime and ceftriaxone, have been shown to inhibit the nuclease activity of hSNM1A *in vitro* at low micromolar efficiency [115]. Structure of each of these compounds bound to hSNM1A have been solved to 1.5 Å and 2.7 Å respectively, and deposited on PDB (PDB ID: 5NZY and 5NZW). Ceftriaxone bound to the metal in the catalytic core of the enzyme and may function by precluding substrate binding and/or enzymatic activity. Conversely, cefotaxime bound in two distinct pockets away from the active site, so the mechanism of action remains unclear. A more recent study showed that nucleoside derivatives containing hydroxamic acids were again able to inhibit the nuclease activity of hSNM1A *in vitro* [116].

## 11 Conclusions and future directions

Twenty years after the identification of *hSNM1A* gene there has been much research into the protein’s structure, function, and cellular role. Although there remains much to be defined, research into the biochemistry and structure of the C-terminal region of hSNM1A has provided insight into its catalytic mechanism and ability to process DNA damage. At the same time, research into specific protein-protein interactions, the repair networks within which SNM1A coordinates and functions, and the role and regulation of PTMs have only provided tantalising glimpses into its cellular role. At this stage, therefore, a clear understanding of hSNM1A’s contribution to genome integrity has not yet fully been realised. Further research into the pathways and mechanisms in which hSNM1A acts in DNA repair will undoubtedly elucidate this further.

Genetic data has provided valuable insight into the role of hSNM1A in the processes of tumourigenesis, treatment outcomes, and survival. Future research will undoubtedly provide insight into the genetic landscape of hSNM1A and may enable the development of inhibitors with therapeutic tractability.

## Funding

Cancer Research UK Programme Award A24759.

## Declaration of Competing Interest

The authors declare that they have no conflicts of interest regarding this work.

## References

[1] Henriques J.A., Moustacchi E. (1980). Isolation and characterization of pso mutants sensitive to photo-addition of psoralen derivatives in Saccharomyces cerevisiae. Genetics.

[2] Henriques J.A., Moustacchi E. (1981). Interactions between mutations for sensitivity to psoralen photoaddition (pso) and to radiation (rad) in Saccharomyces cerevisiae. J. Bacteriol..

[3] Ruhland A. (1981). Isolation of yeast mutants sensitive to the bifunctional alkylating agent nitrogen mustard. Mol. Gen. Genet..

[4] Ruhland A. (1981). A yeast mutant specifically sensitive to bifunctional alkylation. Mutat. Res..

[5] Cassier-Chauvat C., Moustacchi E. (1988). Allelism between pso1-1 and rev3-1 mutants and between pso2-1 and snm1 mutants in Saccharomyces cerevisiae. Curr. Genet..

[6] Brendel M. (2003). Role of PSO genes in repair of DNA damage of Saccharomyces cerevisiae. Mutat. Res..

[7] Wolter R., Siede W., Brendel M. (1996). Regulation of SNM1, an inducible Saccharomyces cerevisiae gene required for repair of DNA cross-links. Mol. Gen. Genet..

[8] Li X., Hejna J., Moses R.E. (2005). The yeast Snm1 protein is a DNA 5’-exonuclease. DNA Repair (Amst).

[9] Tiefenbach T., Junop M. (2012). Pso2 (SNM1) is a DNA structure-specific endonuclease. Nucleic Acids Res..

[10] Demuth I., Digweed M. (1998). Genomic organization of a potential human DNA-crosslink repair gene, KIAA0086. Mutat. Res..

[11] Aravind L. (1999). An evolutionary classification of the metallo-beta-lactamase fold proteins. In Silico Biol. (Gedrukt).

[12] Nagase T. (1995). Prediction of the coding sequences of unidentified human genes. III. The coding sequences of 40 new genes (KIAA0081-KIAA0120) deduced by analysis of cDNA clones from human cell line KG-1. DNA Res..

[13] Callebaut I. (2002). Metallo-beta-lactamase fold within nucleic acids processing enzymes: the beta-CASP family. Nucleic Acids Res..

[14] Hazrati A. (2008). Human SNM1A suppresses the DNA repair defects of yeast pso2 mutants. DNA Repair (Amst).

[15] Buzon B. (2018). Structure-specific endonuclease activity of SNM1A enables processing of a DNA interstrand crosslink. Nucleic Acids Res..

[16] Demuth I., Digweed M., Concannon P. (2004). Human SNM1B is required for normal cellular response to both DNA interstrand crosslink-inducing agents and ionizing radiation. Oncogene.

[17] Lenain C. (2006). The Apollo 5’ exonuclease functions together with TRF2 to protect telomeres from DNA repair. Curr. Biol..

[18] Freibaum B.D., Counter C.M. (2006). hSnm1B is a novel telomere-associated protein. J. Biol. Chem..

[19] van Overbeek M., de Lange T. (2006). Apollo, an Artemis-related nuclease, interacts with TRF2 and protects human telomeres in S phase. Curr. Biol..

[20] Demuth I. (2008). Endogenous hSNM1B/Apollo interacts with TRF2 and stimulates ATM in response to ionizing radiation. DNA Repair (Amst).

[21] Bae J.B. (2008). Snm1B/Apollo mediates replication fork collapse and S Phase checkpoint activation in response to DNA interstrand cross-links. Oncogene.

[22] Mason J.M., Sekiguchi J.M. (2011). Snm1B/Apollo functions in the Fanconi anemia pathway in response to DNA interstrand crosslinks. Hum. Mol. Genet..

[23] Salewsky B. (2012). The nuclease hSNM1B/Apollo is linked to the Fanconi anemia pathway via its interaction with FANCP/SLX4. Hum. Mol. Genet..

[24] Sengerova B. (2012). Characterization of the human SNM1A and SNM1B/Apollo DNA repair exonucleases. J. Biol. Chem..

[25] Moshous D. (2001). Artemis, a novel DNA double-strand break repair/V(D)J recombination protein, is mutated in human severe combined immune deficiency. Cell.

[26] Rooney S. (2002). Leaky Scid phenotype associated with defective V(D)J coding end processing in Artemis-deficient mice. Mol. Cell.

[27] Ma Y. (2002). Hairpin opening and overhang processing by an Artemis/DNA-dependent protein kinase complex in nonhomologous end joining and V(D)J recombination. Cell.

[28] Ma Y., Schwarz K., Lieber M.R. (2005). The Artemis:DNA-PKcs endonuclease cleaves DNA loops, flaps, and gaps. DNA Repair (Amst).

[29] Pannunzio N.R., Watanabe G., Lieber M.R. (2018). Nonhomologous DNA end-joining for repair of DNA double-strand breaks. J. Biol. Chem..

[30] Wang J. (2005). Artemis deficiency confers a DNA double-strand break repair defect and Artemis phosphorylation status is altered by DNA damage and cell cycle progression. DNA Repair (Amst).

[31] Drouet J. (2006). Interplay between Ku, Artemis, and the DNA-dependent protein kinase catalytic subunit at DNA ends. J. Biol. Chem..

[32] Geng L. (2007). Artemis links ATM to G2/M checkpoint recovery via regulation of Cdk1-cyclin B. Mol. Cell. Biol..

[33] Wang H. (2009). Artemis regulates cell cycle recovery from the S phase checkpoint by promoting degradation of cyclin E. J. Biol. Chem..

[34] Betous R. (2018). DNA replication stress triggers rapid DNA replication fork breakage by Artemis and XPF. PLoS Genet..

[35] Zhang X., Richie C., Legerski R.J. (2002). Translation of hSNM1 is mediated by an internal ribosome entry site that upregulates expression during mitosis. DNA Repair (Amst).

[36] Cahill S.T. (2016). Use of ferrous iron by metallo-beta-lactamases. J. Inorg. Biochem..

[37] Bebrone C. (2007). Metallo-beta-lactamases (classification, activity, genetic organization, structure, zinc coordination) and their superfamily. Biochem. Pharmacol..

[38] Palzkill T. (2013). Metallo-beta-lactamase structure and function. Ann. N. Y. Acad. Sci..

[39] Daiyasu H. (2001). Expansion of the zinc metallo-hydrolase family of the beta-lactamase fold. FEBS Lett..

[40] Garau G. (2004). Update of the standard numbering scheme for class B beta-lactamases. Antimicrob. Agents Chemother..

[41] Hejna J. (2007). The hSNM1 protein is a DNA 5’-exonuclease. Nucleic Acids Res..

[42] Allerston C.K. (2015). The structures of the SNM1A and SNM1B/Apollo nuclease domains reveal a potential basis for their distinct DNA processing activities. Nucleic Acids Res..

[43] Newman J.A. (2011). Unusual, dual endo- and exonuclease activity in the degradosome explained by crystal structure analysis of RNase J1. Structure.

[44] Dorleans A. (2011). Molecular basis for the recognition and cleavage of RNA by the bifunctional 5'-3' exo/endoribonuclease RNase. J. Structure.

[45] Li de la Sierra-Gallay I. (2008). Structural insights into the dual activity of RNase. J. Nat Struct Mol Biol.

[46] Mandel C.R. (2006). Polyadenylation factor CPSF-73 is the pre-mRNA 3’-end-processing endonuclease. Nature.

[47] Dronkert M.L. (2000). Disruption of mouse SNM1 causes increased sensitivity to the DNA interstrand cross-linking agent mitomycin C. Mol. Cell. Biol..

[48] Hemphill A.W. (2008). Mammalian SNM1 is required for genome stability. Mol. Genet. Metab..

[49] Ahkter S. (2005). Snm1-deficient mice exhibit accelerated tumorigenesis and susceptibility to infection. Mol. Cell. Biol..

[50] Richie C.T. (2002). hSnm1 colocalizes and physically associates with 53BP1 before and after DNA damage. Mol. Cell. Biol..

[51] Akhter S. (2004). Deficiency in SNM1 abolishes an early mitotic checkpoint induced by spindle stress. Mol. Cell. Biol..

[52] Haaf T. (1995). Nuclear foci of mammalian Rad51 recombination protein in somatic cells after DNA damage and its localization in synaptonemal complexes. Proc Natl Acad Sci U S A.

[53] Long D.T. (2011). Mechanism of RAD51-dependent DNA interstrand cross-link repair. Science.

[54] Ishiai M. (2004). DNA cross-link repair protein SNM1A interacts with PIAS1 in nuclear focus formation. Mol. Cell. Biol..

[55] Hosono Y. (2011). The role of SNM1 family nucleases in etoposide-induced apoptosis. Biochem. Biophys. Res. Commun..

[56] Akhter S., Legerski R.J. (2008). SNM1A acts downstream of ATM to promote the G1 cell cycle checkpoint. Biochem. Biophys. Res. Commun..

[57] Maser R.S. (1997). hMre11 and hRad50 nuclear foci are induced during the normal cellular response to DNA double-strand breaks. Mol. Cell. Biol..

[58] Stracker T.H., Petrini J.H. (2011). The MRE11 complex: starting from the ends. Nat. Rev. Mol. Cell Biol..

[59] Schultz L.B. (2000). p53 binding protein 1 (53BP1) is an early participant in the cellular response to DNA double-strand breaks. J. Cell Biol..

[60] Anderson L., Henderson C., Adachi Y. (2001). Phosphorylation and rapid relocalization of 53BP1 to nuclear foci upon DNA damage. Mol. Cell. Biol..

[61] Muller S. (2001). SUMO, ubiquitin’s mysterious cousin. Nat. Rev. Mol. Cell Biol..

[62] Tallis M. (2014). Poly(ADP-ribosyl)ation in regulation of chromatin structure and the DNA damage response. Chromosoma.

[63] Ahel I. (2008). Poly(ADP-ribose)-binding zinc finger motifs in DNA repair/checkpoint proteins. Nature.

[64] Yang K., Moldovan G.L., D’Andrea A.D. (2010). RAD18-dependent recruitment of SNM1A to DNA repair complexes by a ubiquitin-binding zinc finger. J. Biol. Chem..

[65] Parker J.L., Ulrich H.D. (2009). Mechanistic analysis of PCNA poly-ubiquitylation by the ubiquitin protein ligases Rad18 and Rad5. EMBO J..

[66] Geng L., Huntoon C.J., Karnitz L.M. (2010). RAD18-mediated ubiquitination of PCNA activates the Fanconi anemia DNA repair network. J. Cell Biol..

[67] Dungrawala H. (2015). The replication checkpoint prevents two types of fork collapse without regulating replisome stability. Mol. Cell.

[68] Alabert C. (2014). Nascent chromatin capture proteomics determines chromatin dynamics during DNA replication and identifies unknown fork components. Nat. Cell Biol..

[69] Raschle M. (2015). DNA repair. Proteomics reveals dynamic assembly of repair complexes during bypass of DNA cross-links. Science.

[70] Wang A.T. (2011). Human SNM1A and XPF-ERCC1 collaborate to initiate DNA interstrand cross-link repair. Genes Dev..

[71] Thongthip S. (2016). Fan1 deficiency results in DNA interstrand cross-link repair defects, enhanced tissue karyomegaly, and organ dysfunction. Genes Dev..

[72] Pizzolato J. (2015). FANCD2-associated nuclease 1, but not exonuclease 1 or flap endonuclease 1, is able to unhook DNA interstrand cross-links in vitro. J. Biol. Chem..

[73] Wang R. (2014). DNA repair. Mechanism of DNA interstrand cross-link processing by repair nuclease FAN1. Science.

[74] Zhao Q. (2014). Structural insights into 5’ flap DNA unwinding and incision by the human FAN1 dimer. Nat. Commun..

[75] Andrews A.M. (2018). A senataxin-associated exonuclease SAN1 is required for resistance to DNA interstrand cross-links. Nat. Commun..

[76] Zhang J. (2015). DNA interstrand cross-link repair requires replication-fork convergence. Nat. Struct. Mol. Biol..

[77] Raschle M. (2008). Mechanism of replication-coupled DNA interstrand crosslink repair. Cell.

[78] Muniandy P.A. (2009). Repair of laser-localized DNA interstrand cross-links in G1 phase mammalian cells. J. Biol. Chem..

[79] Williams H.L., Gottesman M.E., Gautier J. (2013). The differences between ICL repair during and outside of S phase. Trends Biochem. Sci..

[80] Enoiu M., Jiricny J., Scharer O.D. (2012). Repair of cisplatin-induced DNA interstrand crosslinks by a replication-independent pathway involving transcription-coupled repair and translesion synthesis. Nucleic Acids Res..

[81] Zheng H. (2003). Nucleotide excision repair- and polymerase eta-mediated error-prone removal of mitomycin C interstrand cross-links. Mol. Cell. Biol..

[82] Richards S. (2005). Triplex targeted genomic crosslinks enter separable deletion and base substitution pathways. Nucleic Acids Res..

[83] Iyama T. (2015). CSB interacts with SNM1A and promotes DNA interstrand crosslink processing. Nucleic Acids Res..

[84] Zhang J., Walter J.C. (2014). Mechanism and regulation of incisions during DNA interstrand cross-link repair. DNA Repair (Amst).

[85] Clauson C., Scharer O.D., Niedernhofer L. (2013). Advances in understanding the complex mechanisms of DNA interstrand cross-link repair. Cold Spring Harb. Perspect. Biol..

[86] Niraj J., Farkkila A., D’Andrea A.D. (2019). The fanconi Anemia pathway in Cancer. Annu Rev Cancer Biol.

[87] McHugh P.J. (2020). XPF-ERCC1: linchpin of DNA crosslink repair. PLoS Genet..

[88] Lopez-Martinez D., Liang C.C., Cohn M.A. (2016). Cellular response to DNA interstrand crosslinks: the Fanconi anemia pathway. Cell. Mol. Life Sci..

[89] Niedernhofer L.J., Lalai A.S., Hoeijmakers J.H. (2005). Fanconi anemia (cross)linked to DNA repair. Cell.

[90] Knipscheer P. (2009). The Fanconi anemia pathway promotes replication-dependent DNA interstrand cross-link repair. Science.

[91] Huang J. (2013). The DNA translocase FANCM/MHF promotes replication traverse of DNA interstrand crosslinks. Mol. Cell.

[92] Fu Y.V. (2011). Selective bypass of a lagging strand roadblock by the eukaryotic replicative DNA helicase. Cell.

[93] Wu R.A. (2019). TRAIP is a master regulator of DNA interstrand crosslink repair. Nature.

[94] Long D.T. (2014). BRCA1 promotes unloading of the CMG helicase from a stalled DNA replication fork. Mol. Cell.

[95] Amunugama R. (2018). Replication fork reversal during DNA interstrand crosslink repair requires CMG unloading. Cell Rep..

[96] Klein Douwel D. (2014). XPF-ERCC1 acts in unhooking DNA interstrand crosslinks in cooperation with FANCD2 and FANCP/SLX4. Mol. Cell.

[97] Abdullah U.B. (2017). RPA activates the XPF-ERCC1 endonuclease to initiate processing of DNA interstrand crosslinks. EMBO J..

[98] Li N. (2020). Cooperation of the NEIL3 and Fanconi anemia/BRCA pathways in interstrand crosslink repair. Nucleic Acids Res..

[99] Semlow D.R. (2016). Replication-dependent unhooking of DNA interstrand cross-links by the NEIL3 glycosylase. Cell.

[100] Huang J. (2019). Remodeling of interstrand crosslink proximal replisomes is dependent on ATR, FANCM, and FANCD2. Cell Rep..

[101] Lake R.J., Fan H.Y. (2013). Structure, function and regulation of CSB: a multi-talented gymnast. Mech. Ageing Dev..

[102] Kohno T. (2006). Association of polymorphisms in the MTH1 gene with small cell lung carcinoma risk. Carcinogenesis.

[103] Madi A. (2018). Pharmacogenetic analyses of 2183 patients with advanced colorectal cancer; potential role for common dihydropyrimidine dehydrogenase variants in toxicity to chemotherapy. Eur. J. Cancer.

[104] Shahi R.B. (2019). Identification of candidate cancer predisposing variants by performing whole-exome sequencing on index patients from BRCA1 and BRCA2-negative breast cancer families. BMC Cancer.

[105] Kikuno R. (2002). HUGE: a database for human large proteins identified in the Kazusa cDNA sequencing project. Nucleic Acids Res..

[106] Fagerberg L. (2014). Analysis of the human tissue-specific expression by genome-wide integration of transcriptomics and antibody-based proteomics. Mol. Cell Proteomics.

[107] Wang X. (2016). A network-pathway based module identification for predicting the prognosis of ovarian cancer patients. J. Ovarian Res..

[108] Laporte G.A. (2020). The role of double-strand break repair, translesion synthesis, and interstrand crosslinks in colorectal cancer progression-clinicopathological data and survival. J. Surg. Oncol..

[109] Wilson M.R. (2019). The human gut bacterial genotoxin colibactin alkylates DNA. Science.

[110] Xue M. (2019). Structure elucidation of colibactin and its DNA cross-links. Science.

[111] Arthur J.C. (2020). Microbiota and colorectal cancer: colibactin makes its mark. Nat. Rev. Gastroenterol. Hepatol..

[112] Helleday T. (2008). DNA repair pathways as targets for cancer therapy. Nat. Rev. Cancer.

[113] O’Connor M.J. (2015). Targeting the DNA damage response in Cancer. Mol. Cell.

[114] Deans A.J., West S.C. (2011). DNA interstrand crosslink repair and cancer. Nat. Rev. Cancer.

[115] Lee S.Y. (2016). Cephalosporins inhibit human metallo beta-lactamase fold DNA repair nucleases SNM1A and SNM1B/apollo. Chem. Commun. (Camb.).

[116] Doherty W. (2019). A hydroxamic-acid-containing nucleoside inhibits DNA repair nuclease SNM1A. Org. Biomol. Chem..

